# Mask and plate: a scalable front metallization with low-cost potential for III–V-based tandem solar cells enabling 31.6 % conversion efficiency

**DOI:** 10.1038/s41598-023-42407-4

**Published:** 2023-09-21

**Authors:** Jörg Schube, Oliver Höhn, Patrick Schygulla, Ralph Müller, Mike Jahn, Gabriele Mikolasch, Marc Steiner, Felix Predan, Jonas Bartsch, Frank Dimroth, Florian Clement, Roman Keding

**Affiliations:** Fraunhofer Institute for Solar Energy Systems (ISE), Heidenhofstraße 2, 79110 Freiburg, Germany

**Keywords:** Applied physics, Semiconductors, Solar cells

## Abstract

Low-cost approaches for mass production of III–V-based photovoltaics are highly desired today. For the first time, this work presents industrially relevant mask and plate for front metallization of III–V-based solar cells replacing expensive photolithography. Metal contacts are fabricated by nickel (Ni) electroplating directly onto the solar cell’s front using a precisely structured mask. Inkjet printing offers low-cost and high-precision processing for application of an appropriate plating resist. It covers the solar cell’s front side with narrow openings for subsequent electroplating. The width of the resulting Ni contacts is as low as (10.5 ± 0.8) µm with sharp edges and homogenous shape. The 4 cm^2^-sized champion III–V-on-silicon triple-junction solar cell with mask and plate front metallization reaches a certified conversion efficiency *η* of (31.6 ± 1.1) % (AM1.5 g spectrum). It performs just as well as the reference sample with photolithography-structured evaporated front contacts, which reaches *η* = (31.4 ± 1.1) %.

## Introduction

High manufacturing costs of III–V-based solar cells limit their application to space and concentrator photovoltaics (PV) today^1^. Even in these fields, the need for low-cost fabrication increases^2^. Price pressure raises to an even higher level in the case of terrestrial PV applications^3^. However, since the tolerable cost per unit area of a solar cell highly depends on module efficiency and application, III–V-based solar cells can have an advantage over state-of-the-art silicon (Si) solar cells^1^. Single-junction devices with a bandgap of 1.12 eV are limited in terms of conversion efficiency *η* to approximately 33 % according to theory^4^. With *η*  = 29.1 %, a gallium arsenide (GaAs) single-junction solar cell holds the world record concerning conversion efficiency for such devices^5^. Multi-junction solar cells can significantly overcome these limitations^6^. In 2018, for a two-terminal III–V-on-silicon (III–V//Si; the double slash symbol indicates a direct wafer bond) triple-junction solar cell, *η*  = 33.3 % (1000 W m^−2^ irradiance, AM1.5 g spectrum) was achieved by using surface-activated wafer bonding and a photonic rear-side structure in the manufacturing process^7^. Two years later, this efficiency was increased to *η*  = 34.1 % (1000 W m^−2^ irradiance, AM1.5 g spectrum)^8^. The gain in *η* was a result of careful adaptations of growing the indium gallium phosphide (GaInP) top cell and band gap tuning of the middle cell^8^. Further developments of the middle cell, namely integration of a gallium indium arsenide phosphide (GaInAsP) absorber, and improved current matching led to the current record conversion efficiency of *η*  = 35.9 % (1000 W m^−2^ irradiance, AM1.5 g spectrum)^9^. Regarding terrestrial applications with a high cost dependency on conversion efficiency, such a high device performance is very desirable, especially for applications with limited space such as, e.g., vehicle-integrated PV^1,8^. However, to pave the way for mass production, strong efforts to reduce manufacturing costs of III–V-based solar cells are needed. This is why several process steps are currently under investigation: the expensive direct wafer bonding process can be replaced, e.g., by direct growth or gluing^10,11^. Metal organic chemical vapor phase epitaxy (MOVPE) costs can be decreased by fast growth, for example^12,13^. However, the realization of low-cost front metallization replacing photolithographically structured evaporated contacts is still missing. A combination with direct growth by fast MOVPE, or alternatively—in the case of pure III–V cells—by reusing the growth substrate, a low-cost metallization could make III–V-based devices suitable for terrestrial applications.

A low-cost metallization has not been shown on highly efficient III–V PV yet: in previous work, the so-called seed and plate approach faced severe challenges when applied to such samples^14^. The limited selectivity resulting in parasitic plating was identified as the main difficulty^14^. To fabricate a metal grid consisting of contact fingers and busbars utilizing this approach, a seed layer is realized using inkjet printing of nano-silver inks. It is printed on the uppermost layer of a III–V-based solar cell precursor, which is a n-type doped GaAs cap layer. Since the seed layer’s cross-sectional area is not large enough to reduce ohmic losses of the device to a tolerable level, it needs to be thickened selectively by galvanic metal deposition, e.g., by using copper electroplating. In fact, the potential difference for electroplating of the silver-based seed layer and the GaAs cap is so small that metal is often not only deposited on the seed but also on the cap layer. Therefore, even at low current densities, the plating process is assessed to be insufficiently selective. Other research groups work on seed and plate metallization of III–V solar cells utilizing copper-based seeds. So far, they do not approach the efficiency of reference cells with photolithographically structured evaporated metal contacts^15^.

A key finding of the work on seed and plate is that galvanic metal deposition directly onto the GaAs cap layer works without the use of (printed) seed layers. This is utilized for a novel approach and the galvanic process is confined to the area where the contact grid is desired. For this purpose, the solar cell is covered with a precisely structured inkjet-printed mask beforehand. After electroplating, the mask is removed wet-chemically unveiling the contact grid. This approach is referred to as mask and plate in the following. A similar approach was already used for (front) metallization of SHJ solar cells^16^. These cells exhibit transparent conductive oxide layers on the front and back that cannot ensure sufficiently homogeneous current distribution for electroplating, which leads to insufficient adhesion of the plated contacts. Therefore, some research groups use physical vapor deposition (PVD) of a conductive seed layer before masking, which is removed wet-chemically after plating^17^. Thereby, finger widths as low as 15 µm can be realized^16^. Note that PVD layers are not used for mask and plate processing on highly doped GaAs cap layers as shown in the present work. This makes processing simpler and cheaper. In fact, both applied techniques, inkjet printing and plating, already proved their industrial potential, scalability, and low-cost potential^18,19^.

The present work focuses on scalable and cost-effective front metallization for III–V-based solar cells in general. It shows its potential on III–V//Si two-terminal triple-junction cells exemplarily. For the first time, the successful application of mask and plate metallization on such devices is demonstrated. The development of this metallization approach from first test structures to the integration into III–V//Si solar cells is presented. Such cells exceed conversion efficiencies of 30 % and, in particular, reach similar efficiencies as the reference devices using photolithography and evaporation processes for metallization. Considering cost and scaling potential, mask and plate has the potential to transform the processing of any III–V-based photovoltaic device.

## Results and discussion

### Mask and plate metallization

**Figure 1:**
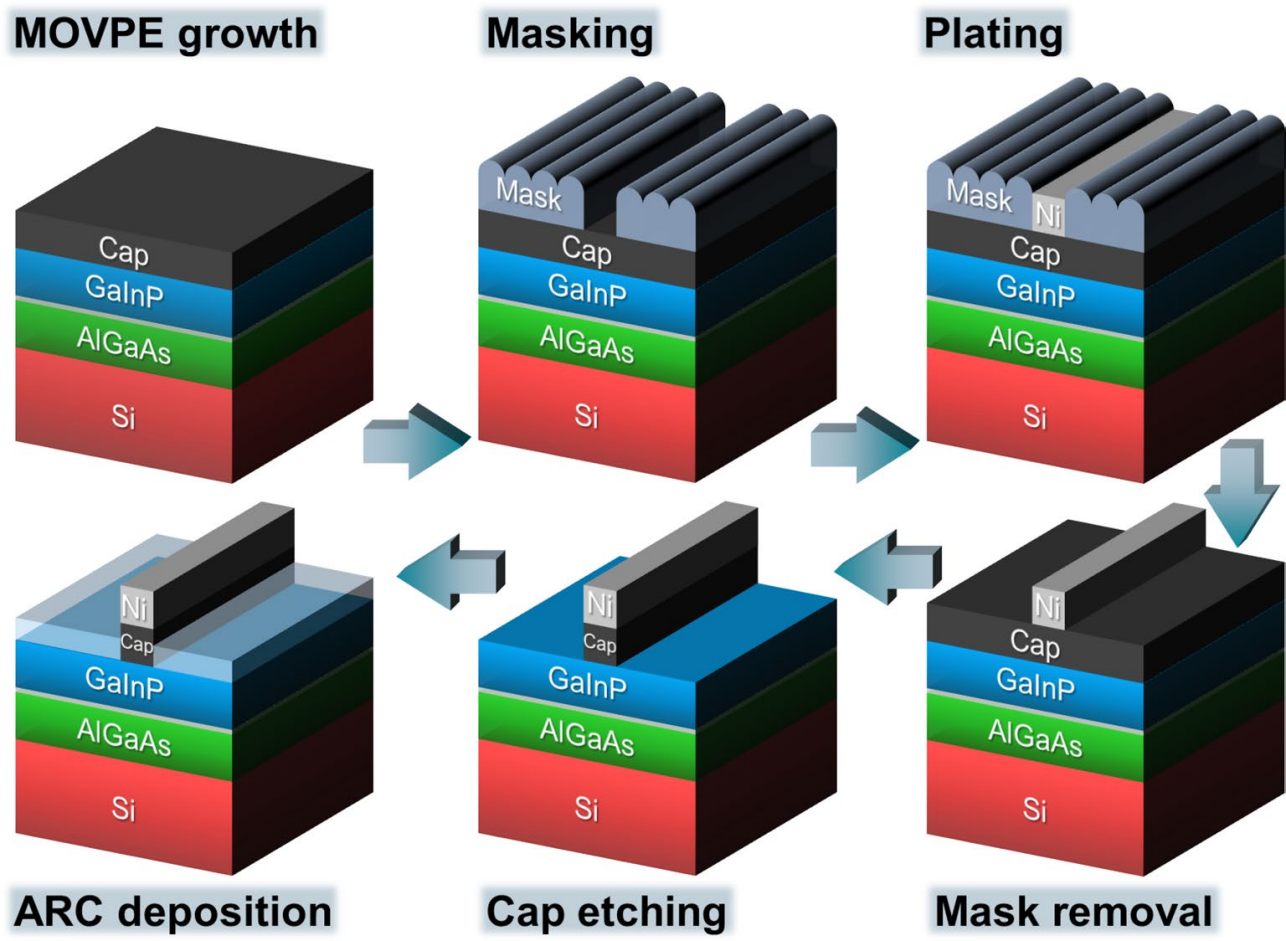
Schematic drawing expressing the placement of mask and plate processing in III–V solar cell manufacturing by taking the example of a III–V//Si device. After MOVPE growth, a mask with precisely structured openings is inkjet-printed on the sample’s front. The wafer’s rear receives a full-area masking, which is ignored in this drawing. Electroplating of front metal contacts is followed by removal of the plating resist. Then, the metal grid acts as a mask for subsequent cap etching. The solar cell’s front side is completed after antireflection coating (ARC) deposition.

In III–V solar cell manufacturing, mask and plate front metallization follows MOVPE growth and replaces both a photolithography and an evaporation process sequence. After front metallization, the cap layer is etched and an antireflection coating (ARC) is deposited on the cell, as Fig. 1 visualizes (see also “Methods” section below). Mask and plate starts with the application of a precisely structured plating resist on the specimen. For this purpose, inkjet printing of a hotmelt ink (also called phase-change ink) meets all requirements, e.g., precision, speed, non-destructiveness, reliability, and robustness of the mask^18^. The ink is melted in the print head and applied to the specimen in a liquid state. It solidifies when in contact with the substrate, which is kept at room temperature. A new two-step printing scheme was developed as part of this work. It allows for the realization of extremely narrow mask openings, which stand out by their sharp edges and great homogeneity in width. The process is described in more detail in the “Methods” section. Moreover, jetting parameters and drop spacing were fine-tuned.

**Figure 2:**
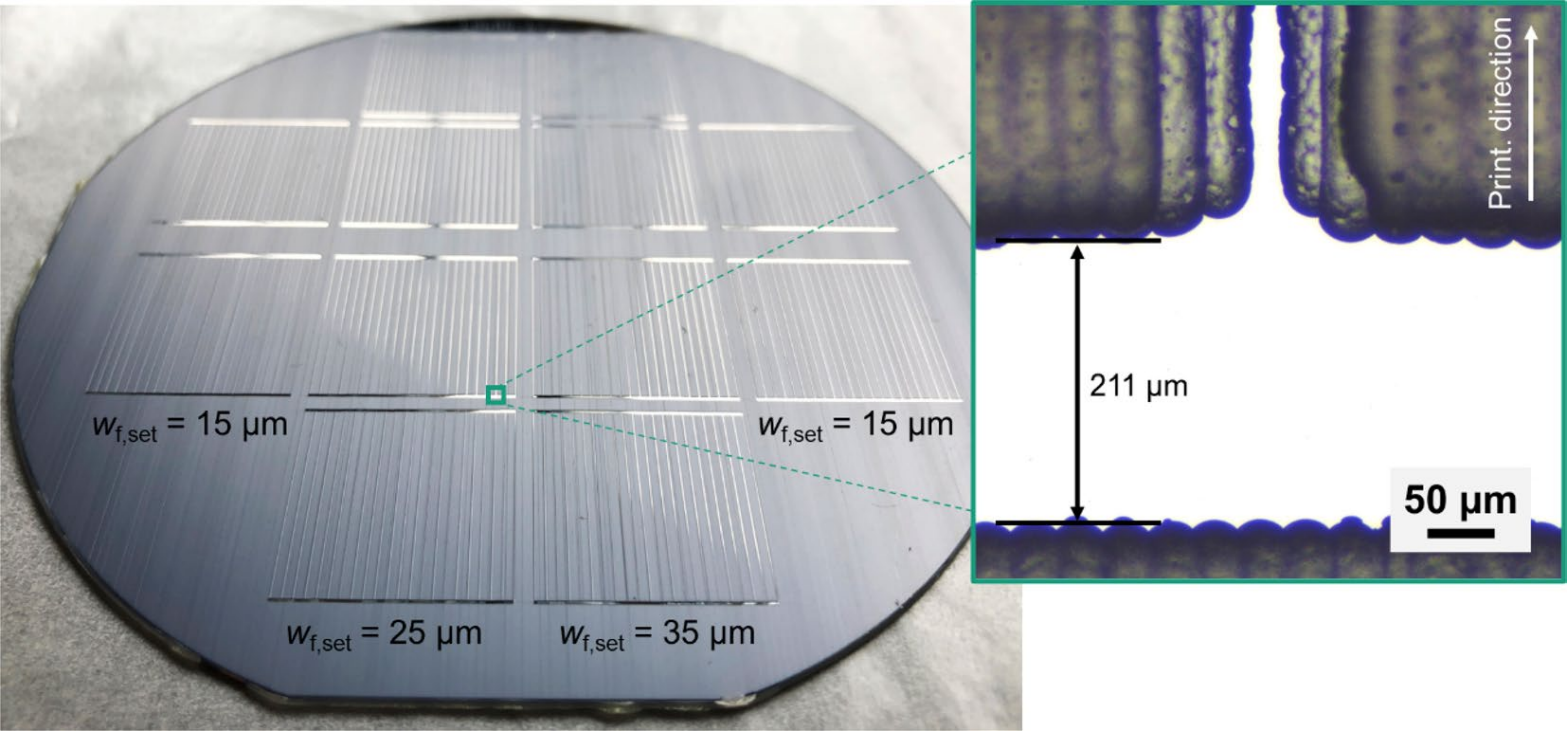
Photograph of a III–V//Si wafer with inkjet-printed plating resist/mask on the front side. The mask is structured to realize twelve front grids for separate 4 cm^2^-sized solar cells. These exhibit two busbars at opposing edges and 22 fingers positioned rectangularly in between a pair of busbars. The mask opening width for the fingers varies from 15 µm (two left and two right cells) over 25 µm (four cells in second column from the left) to 35 µm (four cells in third column from the left). Additionally, a microscope image is shown in a green box. It highlights the transition area where a finger opening meets a busbar opening in the mask.

**Figure 3:**
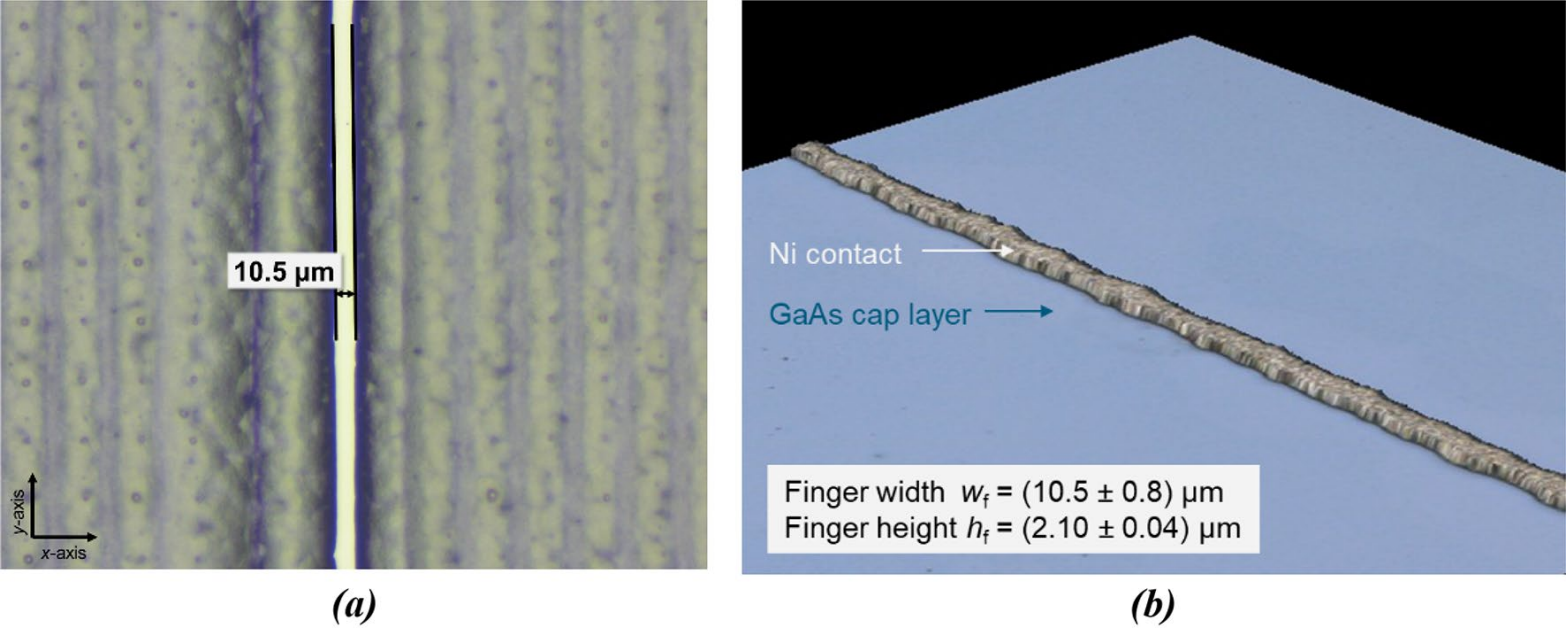
Light microscope image of the smallest mask opening achieved in this work in (**a**). (**b**) presents a 3D confocal microscope image taken after mask and plate processing was completed. The presented error values in (**b**) represent the standard deviations of finger height and finger width for the entire depicted finger segment and was determined using automated image processing.

Figure 2 presents a photograph of a wafer with a diameter of 4’’ after inkjet printing of the hotmelt mask. The printed layout incorporates contact grids for twelve cells as described in detail in the “Methods” section. Opposite to the wafer’s bigger flat, the mask was left open on a small area at the wafer edge for positioning of a contact clamp for subsequent electroplating. Three set mask opening widths *w*_f,set_ for the finger structures, namely 15 µm, 25 µm, and 35 µm, were targeted in this case. Busbar openings were the same in all cases. On other samples, finer mask opening width variations were performed revealing that opening widths of 10.5 µm can be realized without interruptions but very sharp and homogenous shape. This result stands out as it is only about one third of the width that was achieved on III–V//Si solar cells using other up-scalable techniques so far^14^. A microscope image of such a mask opening segment can be found in Fig. 3a. The stripe-like pattern on the left and right side of the mask opening results from inkjet printing of merged lines. Nevertheless, the mask entirely covers the sample next to the desired openings. Using such narrow mask openings, plated contact fingers as exemplarily depicted in Fig. 3b were realized. It demonstrates that fingers with a shading width *w*_f_ (taking all parts of the finger into account contributing to shading-related losses) of (10.5 ± 0.8) µm can be realized using mask and plate processing. In contrast to other plating approaches, such as seed and plate, the metallized area of the sample does not exceed the parts confined by the mask. However, it still needs to be shown that such low feature sizes can be realized at a high yield. Simulations, which are presented further below, show that a further reduction of finger width would be meaningful only if the contact resistivity at the Ni/GaAs contact would be reduced significantly below 1 mΩ cm^2^.

Due to the demand for very narrow mask openings, the transition areas, where fingers and busbars meet, become critical points. Constrictions of the mask openings, which would lead to a significant increase of the solar cell’s resistive losses, must be avoided in these areas. Therefore, drop spacing was fine-tuned at the fingers’ mask openings, where they meet busbars. This ensures great homogeneity in width of the final fingers and, thus, supports the light-generated current flow out of the cell. The microscope image in Fig. 2 exemplarily shows the transition of a mask opening from a finger to a busbar structure. The finger mask opening joins the busbar mask opening under an angle of 90° without significant constrictions, which is important for low-loss current flow in the final device. Furthermore, it is clearly visible in the microscope image that the printed lines, which together form the plating mask, start and stop at the upper and lower edges of the openings confining the busbar, which is orientated perpendicularly to the printing direction. Therefore, these edges are not as homogenous as those of the openings confining fingers, which are orientated along the printing direction. Since the set width of the busbars is one to two orders of magnitude higher than the set width of the fingers, the slightly reduced sharpness of the busbar mask openings is negligible.

After completion of the masking process and a short HF dip, the contact fingers were formed by nickel electroplating into the mask openings. The resistance of the mask against the plating process is obviously of vital importance. E.g., the electrolyte temperature must be below the melting point of the hotmelt ink to prevent detrimental impact on the mask. Moreover, the acidity or basicity of the electrolyte must meet the mask’s specifications. A slightly acidic nickel electrolyte was thus chosen in this work. More details on the plating process can be found in the “Methods” section.

**Figure 4:**
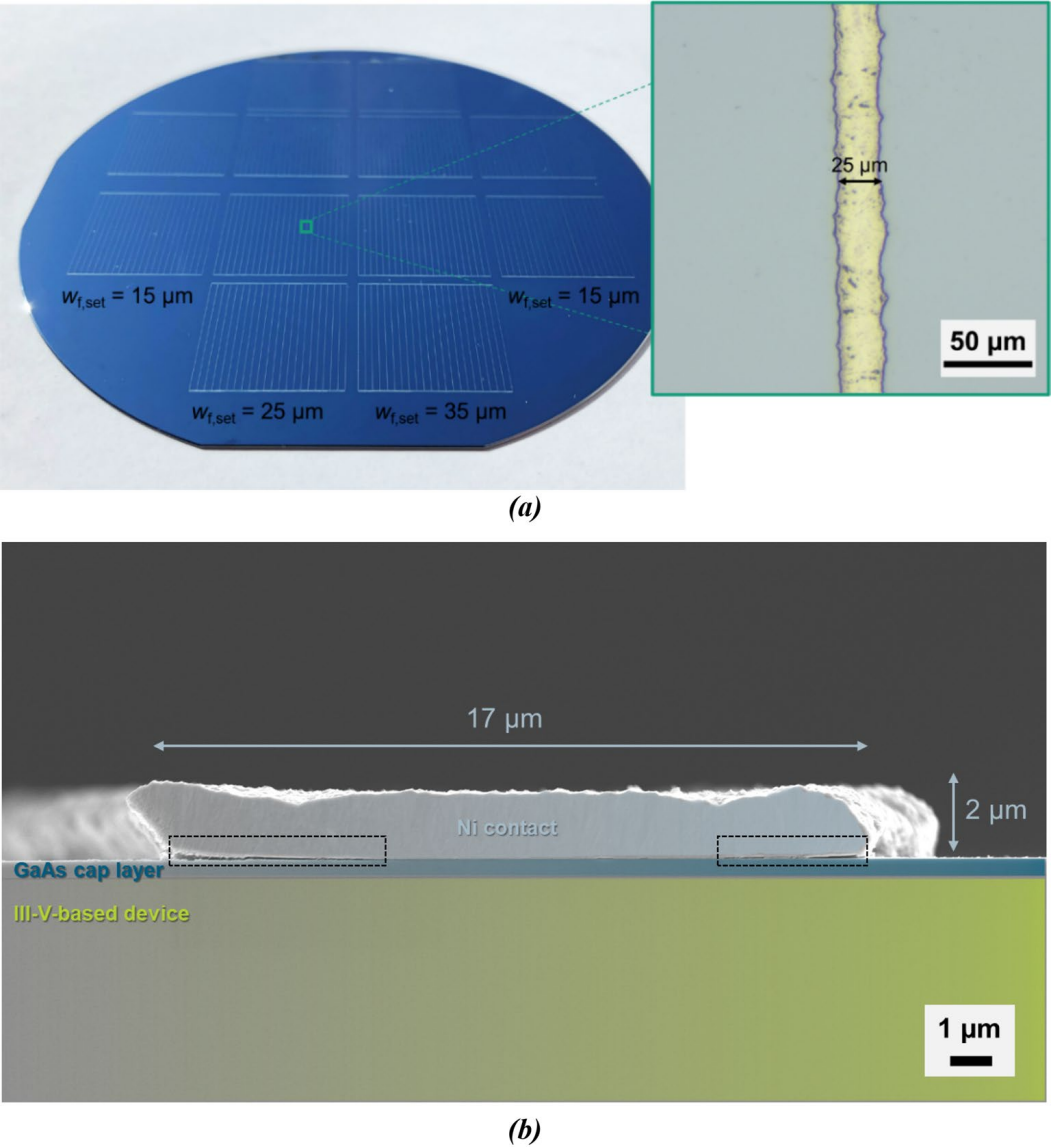
Photo of the same wafer as depicted in Fig. 2 after Ni plating and mask stripping in (**a**). The microscope image in the green box highlights the sharp edges and homogeneity of a finished contact finger. A scanning electron microscope image of a mask and plate Ni contact on a III–V-based substrate with a GaAs cap layer as the uppermost layer is presented in (**b**). The black dashed boxes at the finger’s sides mark finger detachments. For better readability, the Ni contact (light blues), the GaAs cap layer (dark blue) and the III–V substrate (green) are colored.

After electroplating was finished, the mask was removed from the wafer and the metallization process sequence was completed. Figure 4a shows a photo of a wafer with finished mask and plate metallization. Visual inspection and light microscopy conducted after mask removal confirm the high quality of the metal grids as there are hardly any finger defects or interruptions visible across the whole sample area. The plated fingers stand out by their great homogeneity and sharpness at the finger edges (see microscope image in Fig. 4a). This indicates the suitability of the inkjet-printed mask as plating resist because no parasitic plating in masked areas is observed. In other words, the fingers’ shape and width follow exactly those of the mask openings. The fact that the finger edges do not show bleeding-like structures underlines the high quality of this metallization.

Moreover, microstructure analysis of an exemplary mask and plate Ni-based contact was performed based on scanning electron microscopy (SEM). The cross-sectional view of the finger presented in Fig. 4b reveals its very compact microstructure. The finger width of 17 µm nearly equals the set value for the mask opening width of 15 µm. The height of the finger is 2 µm, which could easily be adapted by plating time and electroplating current density adjustments. However, voids are visible on the left and right side of the finger (marked with black dashed boxes) at the interfacial area of nickel finger and GaAs cap layer. It cannot be excluded that they are a result of sample preparation, i.e., sawing and ion polishing, but the fact that other researchers observed similar voids, e.g., at the interface of nickel/copper fingers and ITO layers, suggests that they are not caused by sample preparation only^20^. The voids’ origin is not clear so far. For example, carbon contamination of the mask openings caused by application of the plating resist cannot be ruled out as a reason. Yet, the voids, if not a result of experimental processing, could explain the observed adhesion reduction of the nickel contacts, especially after annealing. It is assumed that the difference in thermal expansion of nickel and GaAs by a factor of two leads to detachment of the metallization departing from such voids at the finger edges^21,22^. Therefore, annealing steps were avoided in solar cell processing after mask and plate processing in this work. The authors are confident that the adhesion reduction is not a fundamental problem of this approach and that it can be fixed by careful optimization. Work is ongoing to tackle this issue, e.g., by physical and chemical pre-conditioning processes aiming for an increase of the contact fingers’ adhesion. In addition to that, it is considered to use other metals than nickel, such as copper or silver for plating. Copper for example allows for highly conductive, low-stress, and ductile metal fingers, as has been demonstrated on silicon-based solar cells already^23^. It is expected that the lower yield strength of copper contacts compared to nickel fingers improves their adhesion even when exposed to post-metallization thermal treatments. Still, the SEM image reveals the great potential of this novel mask and plate process for III–V-based solar cells, as the contact finger’s geometry approaches that of photolithography-structured evaporated contacts.

Although the metallization’s mechanical contact to the GaAs cap layer can be further optimized, the electrical contact is by far sufficient for integration of such metal grids into III–V//Si solar cells at an irradiation of 1000 W m^−2^. Corresponding data characterizing the electrical performance of mask and plate Ni-based contacts can be found in Table 1. The contact resistivity *ρ*_c_ at the Ni finger/GaAs cap layer interface is higher than *ρ*_c_ of the evaporated finger. However, the evaporated fingers were designed for applications with high current flow such as concentrating PV or photonic power converters and the Ni finger/GaAs contact is sufficient for the low current flow in multijunction solar cells in one-sun applications. The modeling presented further below shows this. Measurements of the lateral finger resistance (also referred to as line resistance or finger resistance) and contact geometry analyses demonstrate that the lateral finger resistivity *ρ*_f_ of mask and plate contacts equals the theoretical value *ρ*_f_ = 6.9 µΩ cm for Ni^21^. For the mainly silver-based evaporated contacts, *ρ*_f_ equals the theoretical value *ρ*_f_ = 1.6 µΩ cm for silver^21^. On device level, the higher *ρ*_f_ of mask and plate contacts is compensated by a higher finger cross-sectional area. However, as in the case of *ρ*_c_, higher *ρ*_f_ values of mask and plate contacts can be tolerated in applications with one sun irradiation, which is demonstrated in both the modelling and experimental work presented further below.

**Table 1:** Electrical characterization of mask and plate and reference contacts based on photolithography and evaporation processes.

Metallization	Finger resistivity, *ρ*_f_ (µΩ cm)	Contact resistivity, *ρ*_c_ (mΩ cm^2^)
Mask and plate	6.9^1^	0.6 ± 0.2^2^
Evaporated contacts	1.6^1^	0.001^3^

The error corresponding to the mask and plate finger’s contact resistivity is calculated using Gaussian error propagation.

^1^Value taken from literature^21^.

^2^Value determined based on measurements (see “Methods” section).

^3^Value taken from literature^25^.

Using the data provided in Table 1, network simulations were performed in order to assess the conversion efficiency potential of mask and plate metallization when applied to a III–V//Si tandem solar cell’s front side in comparison with evaporated contacts^24^. To this end, a 20 mm × 20 mm-sized champion device as described in literature with a proven efficiency potential of more than 30 % served as basis^9^. Rectangularly shaped front finger cross sections were assumed. Finger height was chosen to be 3.5 µm both for mask and plate and evaporated contacts. A finger width *w*_f_ of 10 µm was taken as input for mask and plate, *w*_f_ = 8 µm was taken as input for evaporated contacts.

**Figure 5:**
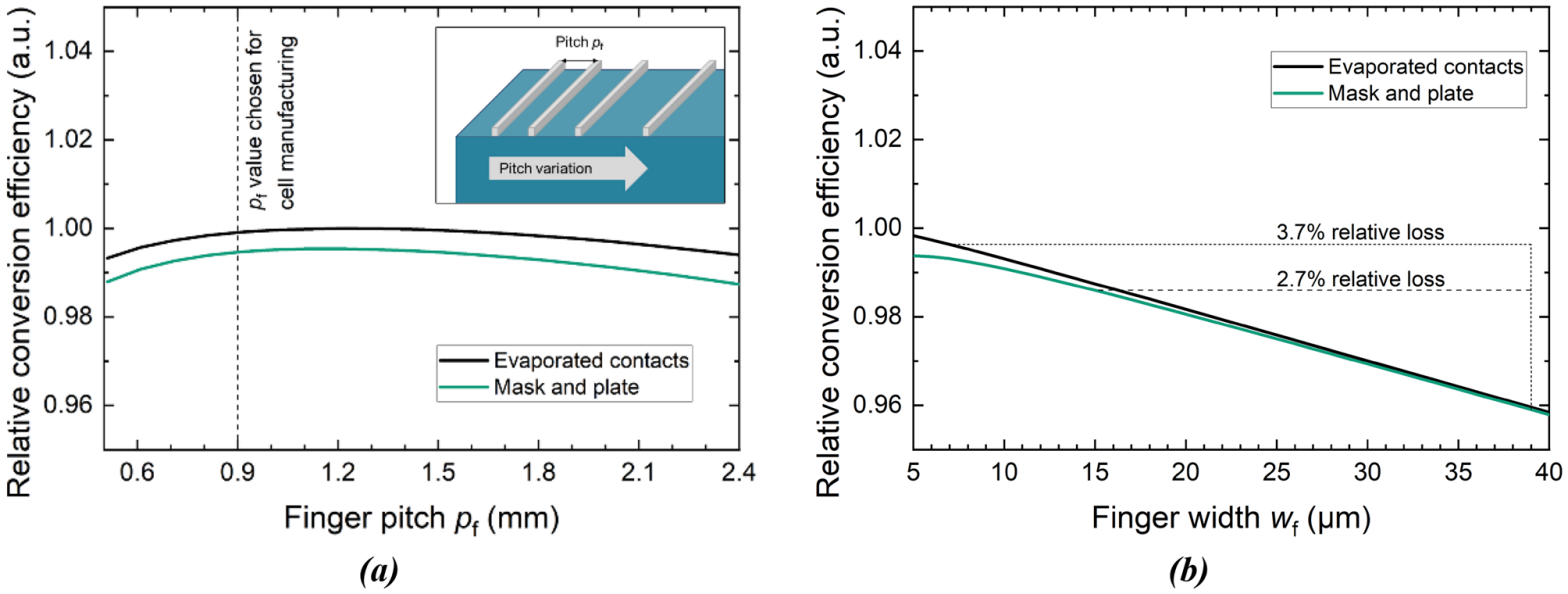
Network simulations of conversion efficiency vs. finger pitch for a III–V//Si solar cell for both, mask and plate as well as evaporated front metallization^24^. Relative conversion efficiency is shown as a function of finger pitch in (**a**). (**b**) shows relative conversion efficiency as a function of finger width at a constant pitch of 0.9 mm.

Figure 5a shows the simulated relative conversion efficiency as a function of the finger pitch *p*_f_ for both mask and plate as well as evaporated front metallization. The data is normalized to the currently highest achieved value of *η*  = 35.9 %^9^. In both cases, the optimum is reached for a finger pitch *p*_f_ = 1.2 mm. For the manufacturing of solar cells, a more conservative value *p*_f_ = 0.9 mm was chosen as described further below (see vertical dashed line in Fig. 5a). A conversion efficiency loss of only 0.4%_rel_ is expected when changing metallization from photolithographically structured evaporated contacts to mask and plate nickel grids while retaining *p*_f_. The difference is mainly driven by higher shading of the mask and plate contacts due to higher finger width. For increasing pitch, the series resistance loss in the solar cell dominates the efficiency loss. Additionally, Fig. 5b shows the correlation of conversion efficiency and shading at a constant pitch of 0.9 mm. The data is used for the evaluation of solar cells further below.

As an interim summary, what can be noted is that using mask and plate, narrow and homogeneous contact fingers with very sharp edges can be produced. This approach utilizes industrially scalable techniques such as inkjet printing and electroplating and is, therefore, promising for mass production of III–V-based PV. Room for improvement is identified regarding the mechanical contact at the Ni/GaAs interface. Still, a sufficiently low contact resistivity is achieved. Modeling reveals a great conversion efficiency potential of III–V-based solar cells with mask and plate front metallization. In the next section, this is confirmed experimentally.

### III–V//Si tandem solar cells with mask and plate front metallization

**Figure 6:**
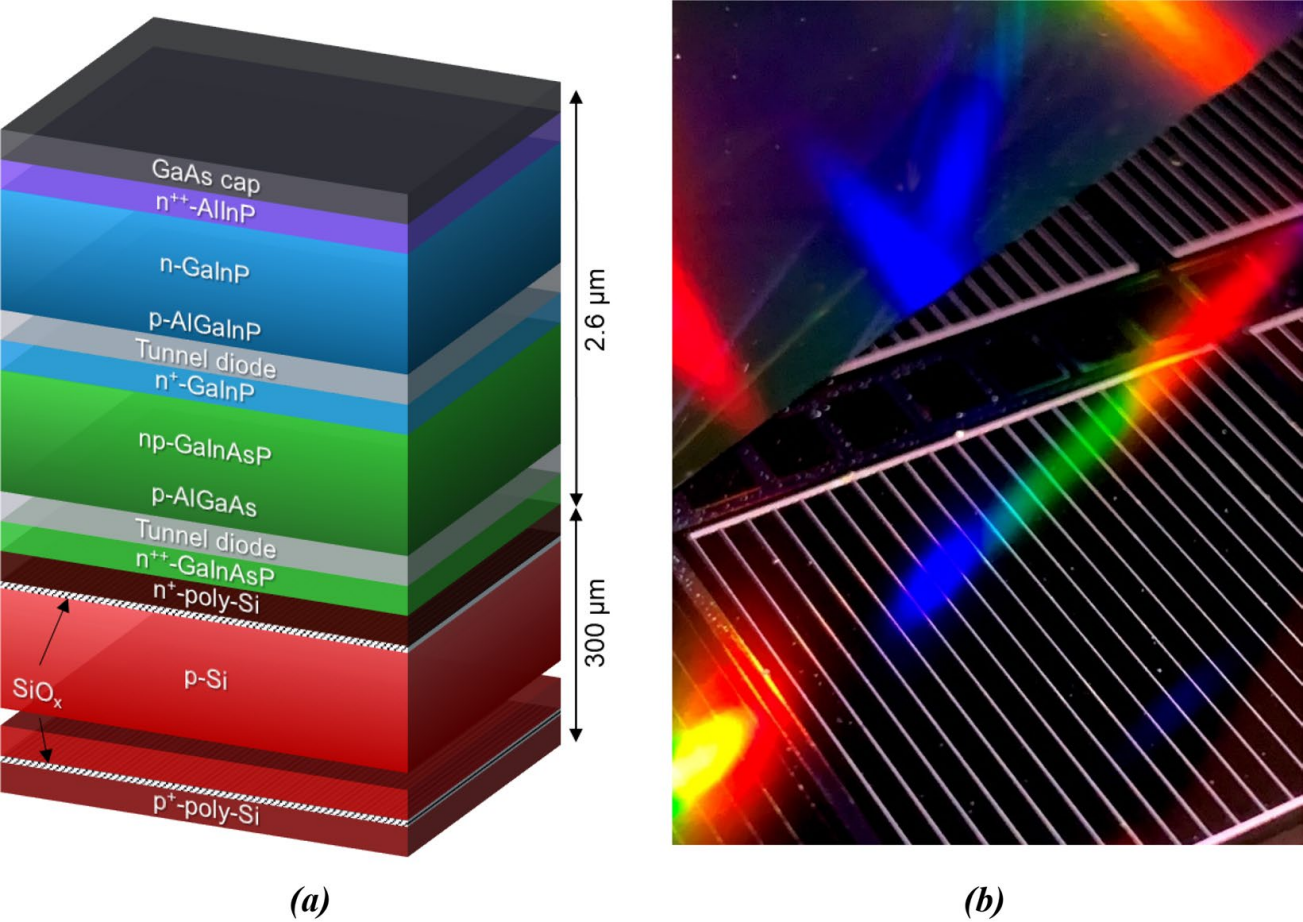
Schematic layer structure of the III–V//Si triple-junction solar cell precursor before metal deposition in (**a**), adapted from literature^9^. The photo in (**b**) shows a mask and plate front grid on top of such a solar cell.

**Figure 7:**
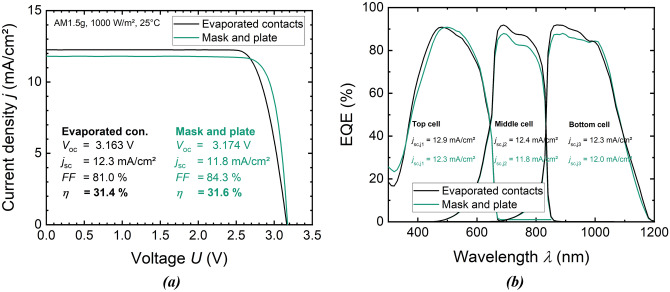
*IV* curves and *IV*-related data corresponding to the two champion solar cells with mask and plate and evaporated front metallization in (**a**). These certified measurements were performed at Fraunhofer ISE CalLab PV Cells. In (**b**) external quantum efficiency data is shown, each one curve represents the cell with evaporated front metallization and the other curve represents the mask-and-plate-metallized cell.

Since the novel mask and plate approach was identified as a very promising metallization method in the previous section, it was integrated into III–V//Si tandem solar cell fabrication. This section focuses on key solar cell results of such devices. The utilized wafer architecture in the state before metallization including the GaAs cap layer is schematically sketched in Fig. 6a. A detailed discussion of this structure can be found in literature^9^. On each 4ʺ-sized wafer, twelve square solar cells with 20 mm edge length were realized. They serve for comparison of mask and plate front metallization with photolithographically structured and evaporated contacts. A photo of a mask and plate cell is depicted in Fig. 6b. The following analysis focuses on the two solar cells achieving the highest efficiency per group in this experiment. The *IV*-related data is presented in Fig. 7a.

The mask and plate champion solar cell reaches a conversion efficiency *η*  = (31.6 ± 1.1) %. This is a remarkable result as this cell performs just as well as the champion cell with evaporated contacts that reaches *η*  = (31.4 ± 1.1) %. The similar *η* values underline the great potential of the mask and plate front metallization for III–V-based solar cells. Moreover, these results are in line with the simulation results predicting a similar performance of the front metallization techniques under comparison (see Fig. 5a).

Although *η* and the open-circuit voltage *V*_oc_ of the two cells presented in Fig. 7a are on the same level, there are significant differences to be discussed in the following. First, a closer look is taken on the short-circuit current density *j*_sc_, which is 11.8 mA cm^−2^ for the mask and plate sample. The reference cell’s *j*_sc_ is 0.4 mA cm^−2^ higher. A deeper understanding of the *j*_sc_ data is gained by the external quantum efficiency (EQE) data provided by Fig. 7b. Both cells reach EQE values of more than 90% in the wavelength range between 470 and 1010 nm. From the short-circuit current densities of the sub-cells *j*_sc,j1_, *j*_sc,j2_, and *j*_sc,j3_ (indices j1–j3 indicate the junctions one to three of the cells from top to bottom), one can observe that *j*_sc,j1_, *j*_sc,j2_, and *j*_sc,j3_ are slightly reduced for the mask and plate cell compared to the reference device. The main reason for this is that shading caused by the front metallization is higher for mask and plate compared to the evaporated contacts. A closer look on key finger geometry parameters is taken in the following. These key parameters are the shading finger width *w*_f_, the finger height *h*_f_, and the finger cross-sectional area *A*_f_. They were investigated directly after metallization and additionally after completing the solar cell manufacturing process. The comparison shows that post-metallization processing has no significant impact on the finger geometry. In this experiment, a cell with the highest target finger width of 35 µm exhibits the highest conversion efficiency. The reason for this is that process steps following the metallization sequence accidentally reduced the performance of the cells in the outer wafer area, where the lowest finger widths were realized (compare Fig. 4a). If contacts with 15 µm target finger width would have been realized on the actual champion mask and plate cell, finger-induced shading would have been reduced by 2.7%_rel_ (compare Fig. 5b).

Table 2 compares the front finger geometries of the two cells under consideration. While the contacts do not significantly differ in height, the mask and plate fingers are (32.0 ± 1.7) µm wider than the evaporated contacts. Obviously, this is reflected in an about eight times higher *A*_f_ value of the mask and plate metallization. The difference in finger width causes 3.7%_rel_ loss in *η* as Fig. 5b shows. Moreover, the mask and plate metal grids contain two busbars in addition to the fingers, whereas the reference grid uses pads instead of busbars. Considering fingers and busbars, the mask and plate front metallization causes ~ 4.5%_rel_ more shading than the reference contacts, which explains the difference in *j*_sc_ of the two cells under comparison.

**Table 2:** Finger geometry data of champion solar cells’ metal grids. These were realized with mask and plate and photolithographically structured evaporation processes.

Metallization	Shading finger width, *w*_f_ (µm)	Finger height, *h*_f_ (µm)	Finger cross-sectional area, *A*_f_ (µm^2^)
Mask and plate	38.7 ± 1.0	3.7 ± 0.4	131.4 ± 14.9
Evaporated contacts	6.7 ± 0.7	3.4 ± 0.1	15.6 ± 1.2

The errors represent the standard deviation.

Going back to the *IV*-related data presented in Fig. 7a, a significant difference of the two cells exists in terms of the fill factor *FF*. It is 3.2%_abs_ higher for the mask and plate cell. By fitting the one-diode equation to the *IV*-curves presented in Fig. 7a, the series resistance *R*_s_ is determined. It is 3.5 Ω cm^2^ for the mask and plate cell as compared to 15.0 Ω cm^2^ for the reference cell. This can be explained partly by the lateral resistances *r*_f_ of the metal contacts, which are calculated using the *A*_f_ and *ρ*_f_ data from Tables 1 and 2, respectively. With *r*_f_ = (10.3 ± 0.8) Ω cm^−1^, the reference contacts induce higher ohmic losses than the mask and plate fingers reaching *r*_f_ = (5.3 ± 0.6) Ω cm^−1^. This results in lower *R*_s_ and, therefore, higher *FF* of the mask and plate cell.

Although *j*_sc_ is limiting the mask and plate cell’s performance, its conversion efficiency clearly exceeds 30 % and is already at the same level as the reference cell. A further improvement of III–V//Si solar cells with mask and plate front metallization can be achieved by simply reducing the shading finger width *w*_f_ and busbar width. Mask and plate contacts with feature sizes of 10 µm are already available today (see Fig. 3b). It is planned to demonstrate the integration of such narrow contacts into solar cell processing in future work, from which a significant improvement of conversion efficiency is expected.

## Conclusion and outlook

For the first time, high-efficiency III–V//Si solar cells with front metallization using the scalable and potentially low-cost mask and plate approach are presented. Following the roadmap towards low-cost III–V-based PV, this is one last piece missing besides, e.g., fast MOVPE. Mask and plate allows for substitution of sophisticated photolithography and evaporation processing by cheaper printing and plating techniques that have proved their scalability potential already. Thereby, similar conversion efficiencies are reached. The champion mask and plate solar cell achieves *η*  = (31.6 ± 1.1) %. This clearly demonstrates the great potential of this metallization approach for III–V//Si solar cells.

Besides that, the present work identifies optimization potential for the mask and plate approach. An even higher performance is expected from grid optimizations. Finger widths of down to 10 µm are already presented on test structure level in this work and are planned to be integrated into high-efficiency solar cells. Even finger widths below 10 µm might be possible by future process optimization but in this case, finger width needs to be fine-tuned together with the contact resistivity at the GaAs interface.

Up to now, mask and plate processing is lab-scale and was not optimized regarding high throughput and costs. In terms of inkjet printing, there is great potential to increase throughput and decrease ink consumption. The following approaches must be tackled in future work: higher throughput can be achieved by inkjet printing speed increase, bidirectional instead of unidirectional printing, and an optimized number of utilized nozzles. Ink consumption can be decreased by increasing drop spacing. Regarding electroplating, the current density could be increased to reduce plating time. Furthermore, finger height has not been fully optimized in terms of efficient metal usage yet.

Besides upscaling and cost reduction, further work on mask and plate front metallization will be dedicated to improved adhesion of metal fingers. This might be achieved by either pre-plating treatments of the sample surface, and/or the galvanic deposition of other metals than Ni, e.g., silver or copper. By exploiting the full potential of mask and plate, the authors expect a conversion efficiency potential for III–V//Si solar cells of more than 35 %.

## Methods

**Figure 8:**
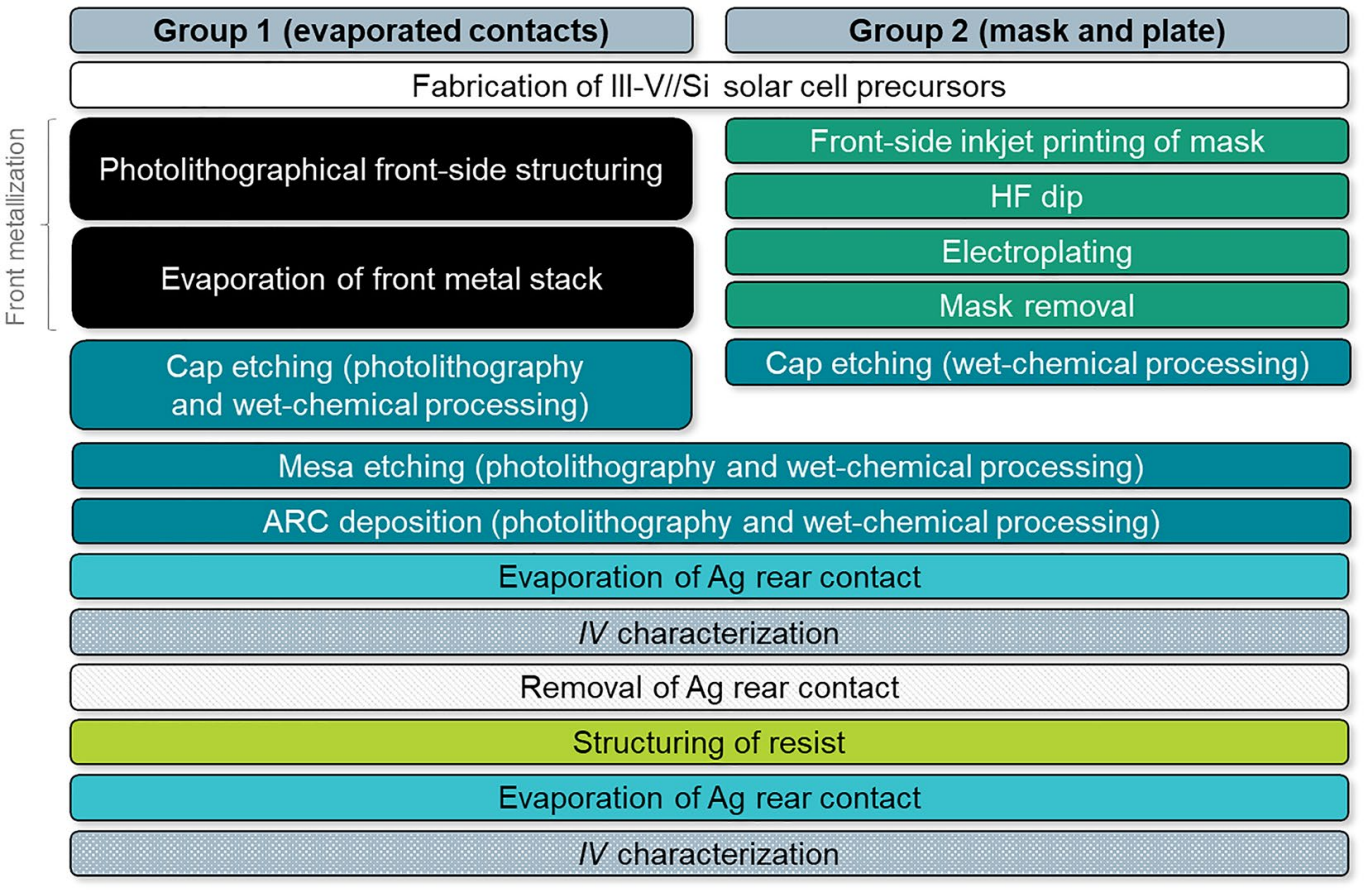
Graphic representation of consecutive process steps utilized for the metallization and post-metallization processing of III–V//Si solar cells.

Figure 8 presents the process steps following III–V//Si solar cell precursor fabrication. Detailed information on key methods used in this work are presented in the following.

### Inkjet printing of mask

Inkjet printing of the plating resist (mask) was performed using a print head integrated into the printing platform LP50 from SÜSS MicroTec (PiXDRO). The print head detects nozzle failure and, therefore, increases the process reliability. For printing, a commercial wax-based and so-called hotmelt (or phase-change) ink, manufactured by Sun Chemical (product code: EMD9325), was heated up to 95 °C in the print head^18^. Then, it was applied in a liquid state to the substrate, which was kept at 25 °C. The ink solidified on the substrate due to cooling below the melting point of 58 °C. The distance between printhead and substrate surface was kept at 500 µm, which results in enhanced precision. Since the process was not optimized for high throughput, printing speed was chosen to be 200 mm s^−1^. First, the wafer’s front side was printed (single-nozzle, unidirectional) starting with pairs of lines confining the area for contact fingers. This approach avoids any relative nozzle deviation and resulting variation in mask openings. Drop spacing was chosen to be 32 µm, which ensures printing of continuous lines on the considered substrate type. Then, the areas between pairs of lines, where no fingers and no busbars were intended, were printed (multi-nozzle printing). The same settings were used for masking the entire rear side of the sample with the plating resist after wafer flipping. Both test structure layouts with set finger width variations and full grid layouts for solar cells as described below were realized.

### Electroplating process and removal of plating resist/mask

After fabrication of the mask, the samples were processed using electroplating. For cleaning reasons, the wafers were dipped in one percent HF solution at room temperature for 30 s beforehand. Then, the wafers were electrically contacted using a clamp (punctual contact). The samples were immersed into the Watts nickel electrolyte (pH = 4.3) so that the electrical contact remained barely above the electrolyte surface. The electrolyte was operated at room temperature. The set current density was chosen to be 1 A dm^−2^ so that 15 min plating time was calculated to be sufficient for nickel deposition.

The plating resist was removed after electroplating. A cascade utilizing isopropanol and acetone was used for this. Afterwards, the sample was rinsed with DI water and dried. Subsequent work on this topic revealed that a cascade using only isopropanol is sufficient.

### Solar cell manufacturing

III–V//Si triple-junction solar cell precursors on 4ʺ wafers were fabricated up to a “metallization-ready” state. The top cells (GaInP/GaInAsP) were grown inverted on a GaAs substrate and joined with the silicon bottom cell by direct wafer bonding^7,9^. After removal of the GaAs substrate, the wafers were equipped with all functional layers except for the front and rear metal contacts. The uppermost layer of the wafers is a 400 nm-thick n-type doped GaAs cap layer exhibiting a charge carrier density *n* = 1 × 10^19^ cm^−3^. A schematic drawing of the full layer stack including the GaInP top cell, GaInAsP middle cell, and Si bottom cell can be found in Fig. 6a.

For the front metallization, one wafer received a mask and plate metallization following the inkjet, electroplating, and mask removal process sequence as described above. The reference wafer received an evaporated front metal grid structured by using photolithography^7^. After metallization processing, the reference wafer with evaporated contacts was annealed. The metallization layout realized on each wafer consists of twelve grids for twelve cells with an edge length of 20 mm. The basic design can be deduced from Fig. 4a. Each grid consists of 22 fingers (finger pitch of 0.9 mm). In the case of mask and plate, three different set widths for the mask’s finger openings were realized: 15 µm, 25 µm, and 35 µm. Furthermore, the mask and plate metal grids include two busbars at opposing cell edges with a set width of 200 µm. The layout of the evaporated metallization is similar to the mask and plate metal grid but exhibits each two pads at opposing cell edges for contacting instead of busbars. The pads are connected with tapered redundancy lines between them which are directed rectangularly to the fingers alongside the cell edge. The set width for the evaporated front contacts was 6 µm for all these grids (no width variation).

After front metallization, the cap layer was etched wet-chemically between the fingers and busbars, or pads. In this process, the metal grids served as etching masks in the case of mask and plate so that the cap layer remained unetched underneath them. In group 1, a photolithography step was necessary. Then, the twelve cells per wafer were electrically isolated from each other by mesa etching into the silicon wafer. This step was followed by evaporation of a double-layer antireflection coating (65 nm Ta_2_O_5_ and 110 nm MgF_2_). The reference wafer with evaporated metal contacts was annealed afterwards to improve the ARC layer’s optics^7^. This was not done with the mask and plate wafer to prevent the metal contacts from potential risk of damage. As a last step, a short HF dip was executed and silver was evaporated on the rear side.

After preliminary *IV*-related characterization of the cells, the silver rear metallization was removed mechanically and the rear-side light trapping was realized. Details about the photonic rear metal structure using nanoimprint lithography can be found in literature^7^. Then, silver was evaporated on the rear side again after a short HF dip.

### Characterization

Characterization focused on *IV*-related solar cell parameters as well as geometrical and electrical characteristics of the metallization. The latter was investigated both after finishing the metallization sequence (before post-metallization processes such as cap etching, etc.) and after *IV* measurements. This was done using light and confocal laser scanning microscopy (Olympus LEXT)^26^. The micrographs were analyzed automatically in terms of finger geometry using inhouse software tools. Selected samples were prepared for scanning electron microscopy^27^. To investigate fingers’ cross sections, they were preconditioned using ion polishing^28^.

The lateral resistances of contact fingers were measured using four-point probe sensing. Either single fingers were measured by assistance of a microscope, or 22 fingers were measured at once with busbar-to-busbar measurements. Using the finger geometry data from confocal microscopy and measurements of finger (segment) lengths, the finger resistivity was calculated^29^. Please note that the measurements were slightly influenced by the conductive cap layer underneath the metal contact. The impact is estimated as not significant, especially in the case of simultaneous measurement of 22 fingers.

For contact resistivity determination, the transfer length method (TLM) was used^30–32^. To this end, samples were sawed into stripes with 10 mm of width. They exhibit equidistant fingers with 0.9 mm pitch, which are contacted by using four-point probe sensing according to TLM.

*IV*-related parameters of all fabricated cells were characterized by a class-A solar simulator with a xenon lamp. Based on this screening, selected cells were characterized in detail at the Fraunhofer ISE CalLab PV Cells. By means of a grating monochromator setup with adjustable bias voltage and bias spectrum, external quantum efficiencies were measured^33^. *IV* characteristics were determined at standard testing conditions (IEC 90604-3, ed. 2 with 1000 W m^−2^) under a spectrally adjustable solar simulator with one xenon lamp and two halogen lamp fields^34^. They were adjusted in intensity independently of each other to generate the same current densities in each sub-cell as under illumination with the AM1.5 g spectrum. An aperture mask was used for these measurements with an open area of 3.987 cm^2^. The solar cell results thus refer to aperture area measurement including shading by fingers, busbars, or pads. More details about the measurement setup and procedure can be found in literature^7^.

## Data Availability

The data that support the findings of this study are available from the corresponding author upon reasonable request.
